# A period of structural plasticity at the axon initial segment in developing visual cortex

**DOI:** 10.3389/fnana.2014.00011

**Published:** 2014-03-11

**Authors:** Annika Gutzmann, Nursah Ergül, Rebecca Grossmann, Christian Schultz, Petra Wahle, Maren Engelhardt

**Affiliations:** ^1^CBTM, Medical Faculty Mannheim, Institute of Neuroanatomy, Heidelberg UniversityHeidelberg, Germany; ^2^AG Developmental Neurobiology, Faculty of Biology and Biotechnology, Ruhr-UniversityBochum, Germany

**Keywords:** visual cortex, plasticity, axon initial segment, dark rearing, ankyrin-G

## Abstract

Cortical networks are shaped by sensory experience and are most susceptible to modifications during critical periods characterized by enhanced plasticity at the structural and functional level. A system particularly well-studied in this context is the mammalian visual system. Plasticity has been documented for the somatodendritic compartment of neurons in detail. A neuronal microdomain not yet studied in this context is the axon initial segment (AIS) located at the proximal axon segment. It is a specific electrogenic axonal domain and the site of action potential (AP) generation. Recent studies showed that structure and function of the AIS can be dynamically regulated. Here we hypothesize that the AIS shows a dynamic regulation during maturation of the visual cortex. We therefore analyzed AIS length development from embryonic day (E) 12.5 to adulthood in mice. A tri-phasic time course of AIS length remodeling during development was observed. AIS first appeared at E14.5 and increased in length throughout the postnatal period to a peak between postnatal day (P) 10 to P15 (eyes open P13–14). Then, AIS length was reduced significantly around the beginning of the critical period for ocular dominance plasticity (CP, P21). Shortest AIS were observed at the peak of the CP (P28), followed by a moderate elongation toward the end of the CP (P35). To test if the dynamic maturation of the AIS is influenced by eye opening (onset of activity), animals were deprived of visual input before and during the CP. Deprivation for 1 week prior to eye opening did not affect AIS length development. However, deprivation from P0 to 28 and P14 to 28 resulted in AIS length distribution similar to the peak at P15. In other words, deprivation from birth prevents the transient shortening of the AIS and maintains an immature AIS length. These results are the first to suggest a dynamic maturation of the AIS in cortical neurons and point to novel mechanisms in the development of neuronal excitability.

## Introduction

Visual cortex development is a highly complex, distinctly regulated process, which is based on the intricate play of intrinsic, genetic and environmental determinants such as growth factors or neuronal activity (Berardi and Maffei, 1999; Hensch, 2005; Fagiolini et al., 2009). A key factor involved in this process is visual experience, which shapes retinal, subcortical and cortical circuits during critical periods characterized by extensive cellular plasticity as evident in the formation of dendritic and axonal arbors, determination of neurochemical phenotypes, soma size, synaptogenesis and synaptic plasticity. Ultimately, these structural modifications lead to the emergence of global alterations such as ocular dominance shifts (Hubel and Wiesel, 1963; Berardi et al., 2000). In mice, the developmental sequence is as follows: first, a precritical period is characterized by initial circuit formation independent of visual experience (until P19), second, the CP with the highest sensitivity to input imbalances peaks around P28, and third, circuit consolidation and closure of the CP (around P35) occurs.

While the effects of spontaneous and sensory-driven activity for somatodendritic plasticity have been well characterized (Levelt and Hubener, 2012), the activity-induced reorganization of axonal domains has been less studied. Recently, Cheetham et al. reported that sensory experience can induce fine-scale remodeling of axons, expressed by changes in the turnover rate of axonal varicosities on layer II/III axons in the somatosensory cortex after whisker trimming (Cheetham et al., 2008). The same authors also reported that in deprived somatosensory cortex, local excitatory connections between layer II/III pyramidal neurons can be lost (Cheetham et al., 2007). Furthermore, it was shown that axonal sprouting of long-range lateral projections accompanies remodeling of cortical functional architecture in a model of binocular retinal lesions in cat (Darian-Smith and Gilbert, 1994). Thus, sensory experience can have a significant impact on axons depending on how the input is altered.

In the context of axonal plasticity, the AIS has recently been implicated as a dynamic structure. The AIS is an electrogenic microdomain essential for action potential (AP) initiation (Rasband, 2011) and is a crucial determinant of cellular excitability (Grubb and Burrone, 2010; Kuba et al., 2010). In pyramidal cortical neurons, the AIS receives synaptic input from chandelier interneurons, with the number of contacts varying depending on age and location of projection target of the cells (Inda et al., 2007). In general, the AIS is characterized by a highly specific set of molecular components. The master scaffolding protein ankyrin-G (ankG) is involved in clustering of ion channels, cell adhesion and signaling molecules (Zhou et al., 1998; Bennett and Chen, 2001; Bennett and Healy, 2009). Three major isoforms of ankyrin proteins have been described, of which two, ankG and ankB, have been shown to be of fundamental importance for the structural and functional maturation of electrogenic axon domains (Bennett et al., 1997; Rasband, 2011; Engelhardt et al., 2013). AnkG binds to the actin cytoskeleton via βIV spectrin (Rasband, 2010), which results in the establishment of a highly stable protein complex (Galiano et al., 2012). Functionally, ankG is a key determinant of neuronal polarity (Hedstrom et al., 2008; Sobotzik et al., 2009). Recent studies have demonstrated that the AIS is a dynamically regulated structure under physiological and pathophysiological conditions (Grubb and Burrone, 2010; Kuba, 2010; Hinman et al., 2013).

Here, we address the question whether the AIS in developing visual cortex of mice undergoes a period of dynamic structural development similar to that known from the somatodendritic domain. We document for the first time the structural development of the AIS in visual cortex neurons from the embryonic period to adulthood. Furthermore, we show that visual experience is an important determinant for AIS maturation and that the AIS can undergo phases of plasticity *in vivo* depending on sensory inputs. Overall, our study demonstrates that visual input influences structural characteristics of cortical AIS and hence possibly alters neuronal excitability *in vivo*.

## Materials and methods

### Animals

All animal protocols in this study were approved by the Heidelberg University, Medical Faculty Mannheim Animal Research Board as well as the State of Baden-Württemberg, Germany and were conducted in accordance with Heidelberg University Guidelines on the Care of Laboratory Animals. Animals of mixed gender from the C57BL/6JRj strain (Janvier Labs, France) were maintained with food and water *ad libitum* on a regular 12 h light/dark cycle (except in deprivation studies as outlined below).

### Developmental study

A total of 6 brains were analyzed in each age group (E12.5, 14.5, 20.5, P1, 3, 7, 10, 15, 21, 28, 35, >P55, >P180). Over 100 AIS were examined in layers II/III and V, respectively, in the primary visual cortex of each brain. In age groups E14.5 and E20.5, cells throughout the marginal zone, cortical plate and subplate were counted (less than 100). As a control, AIS of layer II/III cortical neurons from a non-sensory cortex (cingulate cortex) were analyzed as well. Here, a total of 3 brains from P7, P15, P28, and >P180 animals were used.

### Visual deprivation

Six mice at various ages were kept in completely dark cages with food and water *ad libitum* for 1 week (P8–15, P21–28) and 2 weeks (P14–28, P21–35). Total absence of light was controlled for by exposure of photographic paper placed in the cages. Additional groups of six mice each were reared in complete darkness (P0–7, P0–15, P0–28, P0–35). All control and experimental groups are referenced in Table 1. Analysis then proceeded as described below immediately after that period of visual deprivation. Data from the developmental study also served as controls for the deprivation.

**Table 1:** **Experimental and control groups used in the current study with indication of age of animal, period of visual deprivation and treatment of tissue for immunofluorescence**.

**Control**	**Visual deprivation**	**Fixation (1% PFA)**
E12.5, 14.5, 20.5		Immersion
P1, P3, P7	1 week (P0–7)	Immersion
P10		Perfusion
P15	From birth (P0–15)	Perfusion
P21		Perfusion
P28	From birth (P0–28)	Perfusion
	1 week (P21–28)	
	2 weeks (P14–28)	
P35	From birth (P0–35)	Perfusion
	2 weeks (P21–35)	
>P55, >P180		Perfusion

E, embryonic; P, postnatal; PFA, paraformaldehyde.

### Immunohistochemistry

Brains of animals of age groups E12.5 to P7 were fixed overnight by immersion in 1% paraformaldehyde (PFA, in 0.1 M phosphate buffer, pH7.4) at 4°C and sucrose treatment as outlined below was applied. For the embryonic ages, the skull was opened along the midline to allow for penetration of fixative. For animals at P1–7, brains were removed from the skull and fixed by immersion. Brains from animals P10 and older were exsanguinated with 0.9% NaCl under deep anesthesia and perfusion-fixed with ice-cold 1% PFA. After overnight immersion in 1% PFA at 4°C, brains were cryoprotected by sequential incubation in 5% (2 h), 10% (2 h), and 30% (overnight) sucrose. Brains were trimmed to a block including visual cortex and embedded in Tissue Tek® (Sakura Finetek). Double and triple immunofluorescence was performed on free-floating 30 μm thick cryostat sections for age groups P7 and older. Sections (20 μm) from younger age groups were stained directly on slides. In a control experiment, no difference in AIS length between free floating and mounted sections was observed. A detailed description of all antibodies used in this study is given in Table 2. Immunostaining was performed according to a previously published method (Engelhardt et al., 2013). Omitting the primary antibody completely abolished all staining's. Previously conducted controls are detailed in Table 2.

**Table 2:** **Specification of antibodies with indication of catalog number, clone, working dilution, previously conducted controls, sources and references where available, and the corresponding figure in the manuscript**.

**Antibody catalog No. Clone/type**	**Dilution in IF and WB**	**Reported specificity**			**Source references**	**Figures**
		**KO**	**IHC**	**WB**		
α/β *tubulin* (rb) #2148	WB: 1:1000		X	X	Cell signaling, Frankfurt, Germany Marini et al., 2006	Figures 5C,E,F
*Ankyrin-G* (rb) sc-28561 H-215	IF: 1:500 WB: 1:300	X	X	X	Santa Cruz, Heidelberg, Germany Engelhardt et al., 2013	Figures 1A,D, 2B,D–G, 3B–C_1_, 5A–C,E, S1A,C,E
*Ankyrin-G* (ms) 73-146 N106/36	IF: 1:500	X	X	X	UC Davis/NIH NeuroMab Facility, CA, USA Engelhardt et al., 2013	Figures 2C, S1A,C,E, S2A,C,E
β*IV spectrin* (rb) *amino acids 2237–2256 of human* β*IV spectrin*	IF: 1:500	X	X	X	Selfmade Berghs et al., 2000	Figures 1B, S2A,C,E
*Myelin basic protein* (rt), ab7349, clone 12	IF: 1:1000 WB: 1:15000			X	AbCam, Cambridge, UK Engelhardt et al., 2013	Figures 5A,B,E
*NeuN* (ms) MAB377, clone A60	IF: 1:500		X	X	Millipore, Temecula, USA Engelhardt et al., 2007	Figures 1A,B, 3B,C_1_
*panNa_V_* (ms) S8809 clone K58/35	IF: 1:500		X	X	Sigma, Saint Louis, MO, USA Rasband et al., 1999	Figures 1C,D, 5F
*reelin* (ms) MAB5364, clone G10	IF: 1:500		X	X	Millipore, Billerica, USA Doehner et al., 2012	Figure 2D

IF, immunofluorescence; IHC, immunohistochemistry; IP, immunoprecipitation; KO, lack of signal in knock out animals; WB, western blot; rb, rabbit; ms mouse; rt, rat.

### Western blot

A total of three mice per age group (P1 through P180) were used. Animals were transcardially perfused with ice-cold 0.9% NaCl. The occipital pole of all brains was prepared as a block using a brain matrix slicer (Zivic Instruments). An increase in ankG signal was to be expected for older animals due to the onset of myelination and the appearance of ankG-positive nodes of Ranvier. Therefore, protein lysates were prepared as follows. Regions with visual cortex from animals P21 and older were cut into 1.0 mm thick coronal slabs which were transferred to 35 mm wells and submersed in ice-cold phosphate buffer. Under microscope guidance, the upper and lower layers of primary visual cortex were carefully isolated and collected separately. The white matter was eliminated from all samples. Visual cortex from animals at P1–15 was prepared without upper and lower layer distinction. Tissue was lysed in chilled buffer (20 mM Tris pH 7.5, 500 mM NaCl, 0.5% CHAPS, 5 mM EDTA in Aqua dest.) supplemented with phosphatase and protease inhibitor (Roche) followed by ultrasonic disruption. To determine protein concentration, a modified Bradford's assay was performed (Roth). SDS PAGE (8%) with a discontinuous buffer system was utilized, and 10 μl (1 μg protein/μl) were loaded along with a protein standard (Biorad). After transfer to PVDF membrane (Immobilon®-P, pore size: 0.45 μm), membranes were blocked and incubated overnight at 4°C with primary antibodies diluted in blocking buffer as outlined in Table 2. After washing, membranes were incubated in secondary antibody (polyclonal goat, anti-rabbit and anti-rat immunoglobulins/horseradish peroxidase, 1:2000) for 90 min at 4°C. Protein blots were processed using the ECL detection solution (Thermo Scientific) and either developed for 5 min on film (Raymed Imaging AG) for qualitative analysis, or were scanned with Fusion Capt Advanced Software (Fusion Solo, Peqlab, Germany) for semi-quantitative analysis. Relative band intensities were determined densitometrically for the 270 kDa ankG band using ImageJ Western blot quantification (NIH, USA). AnkG protein was normalized to the internal loading control (α/β Tubulin). Normalized levels of ankG are expressed relative to the normalized ankG expression at P7, which was set to 1. Wilcoxon rank sum test for non-parametric samples was utilized for statistics (^*^p ≤ 0.05).

### Confocal analysis

Confocal analysis was carried out on a C1 Nikon confocal microscope with a 60x (oil immersion, 1.4 NA) objective. To increase the number of in-focus immunoreactive structures, stacks of images were merged into a maximum intensity projection and saved as tiff format. Thickness of single optical sections was 0.5 μm in stacks of 10–20 μm total depth. Images for qualitative analysis were evaluated and enhanced for contrast in Adobe Photoshop CS5 (Adobe Systems).

### Image and AIS length analysis

For image analysis, a self-written macro in Fiji (ImageJ) was used. Images were preprocessed by histogram stretching and a sharpening filter to enhance edges, using the standard settings of the program. An AIS was only included in the analysis if the ankG immunosignal identified a single entity within the recorded stack and the corresponding soma was clearly distinguishable. AIS were plotted overlapping at both ends into the somatic domain as well as into the distal section of the axon. This line was then straightened automatically and data was saved as a diagram plotting number of pixels vs. intensity of ankG staining for each AIS as an Excel (Microsoft) file. A self-written macro in Visual Basic for Applications (VBA, Excel, Microsoft) served to read out the length of each AIS from the previously recorded raw data file. The AIS length cut-off was set at 10% of the individual maximum intensity in each data table to avoid taking background noise into account. Length in pixels was converted into μm based on the microscope's calibration (0.21 μm/pixel at 600x). All AIS longer than 10 μm were included into statistical analysis.

**Figure 1:**
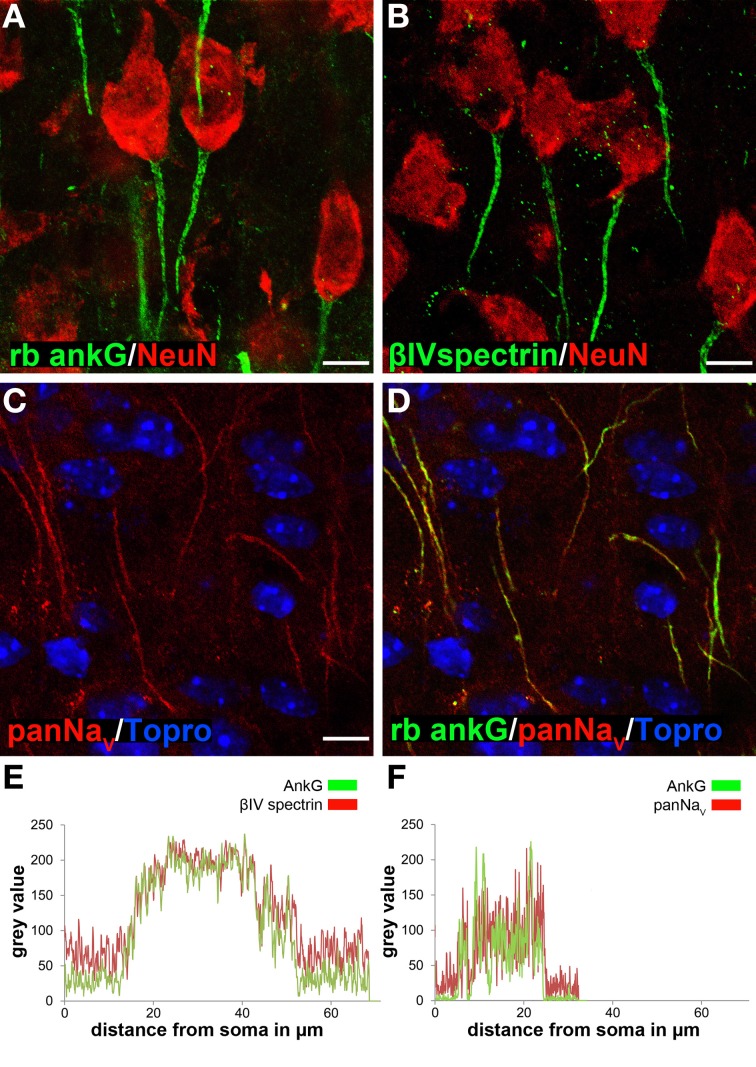
**Ankyrin-G as a reliable marker for AIS length analysis in cortical neurons**. AIS were specifically stained using various markers. **(A)** AnkG (green) and NeuN (red) in layer II/III neurons in a P35 animal. **(B)** βIV spectrin (green) and NeuN (red) in layer II/III neurons in a P35 animal. **(C)** PanNa_*V*_ (red) in layer II/III neurons in a P15 animal. **(D)** Merged image of C and ankG (green) with TO-PRO^©^-3 (blue) as nuclear stain, showing complete overlap of ankG and panNa_*V*_ signals. **(E)** Fluorescence intensity profile for AIS markers ankG (green) and βIV spectrin (red) in an example of a long AIS in a P15 animal, indicating signal overlap for the same AIS distance from the soma. **(F)** Fluorescence intensity profile for ankG (green) and panNa_*V*_ (red), here in an example of a short AIS (P15). Again, signals overlapped with both markers. Scale bars **(A–D)** = 20 μm.

In prior studies, two physical attributes have been used to measure structural changes in the AIS: position with respect to the soma and length. Several studies reported AIS position shifts under developmental, pharmacological, and pathological conditions (Grubb and Burrone, 2010; Galiano et al., 2012; Harty et al., 2013). In our hands, careful tracing of the soma boundary and the proximal beginning of the AIS indicated no gap in cases where the soma and the AIS appear as belonging to the same cell (see Figure 1). Therefore, we concentrated on AIS length, which has been proven as a robust parameter of structural changes (Kuba et al., 2010; Duflocq et al., 2011; Hinman et al., 2013).

### Statistical analysis

Mean values and standard deviations were calculated in Excel (Microsoft) and plotted in Sigma Plot 12.0 software (Systat Software GmbH). Wilcoxon rank sum test for non-parametric samples was carried out in R (http://www.r-project.org). Diagrams in Figures 3, 6 show the mean of all 6 animals from each age group, while the diagram in Figure 4 shows the mean of three animals per age group as control. Error bars indicate inter-individual standard deviation between the 6 and 3 means, respectively, of each group. Asterisks indicate significant differences (^*^p ≤ 0.05, Wilcoxon rank sum test).

## Results

### Determination of appropriate antibodies for analysis of AIS development

First, we determined the most suitable antibody for immunofluorescence by comparing different species-derived ankG, βIV spectrin and voltage-gated sodium channel antibodies as previously described (Rasband, 2010). All tested antibodies produced reliable and specific AIS signals especially in early postnatal animals (Figures 1A–D, S1A,C,E, 2A,C,E). Length measurements of the AIS using both a rabbit polyclonal and mouse monoclonal anti-ankG antibody as well as βIV spectrin showed no difference between the respective markers in terms of specificity, reliability and fluorescence intensity overlap (Figures 1E, S1B,D,F, 2B,D,F). Double-staining of ankG and sodium channels also showed signal overlap (panNa_*V*_, Figure 1F). Based on these findings, the rabbit polyclonal ankG antibody was chosen as the appropriate AIS marker for further quantitative analysis because (1) it precedes βIV spectrin expression at the AIS (Dzhashiashvili et al., 2007) and (2) the fluorescence intensity of panNa_*V*_ immunostaining appeared to be reduced in animals older than P21. Interestingly, we observed a clear difference (see below) in cellular distribution of ankG-like immunoreactivity when comparing the commercially available monoclonal and polyclonal ankG antibodies (Table 2).

**Figure 2:**
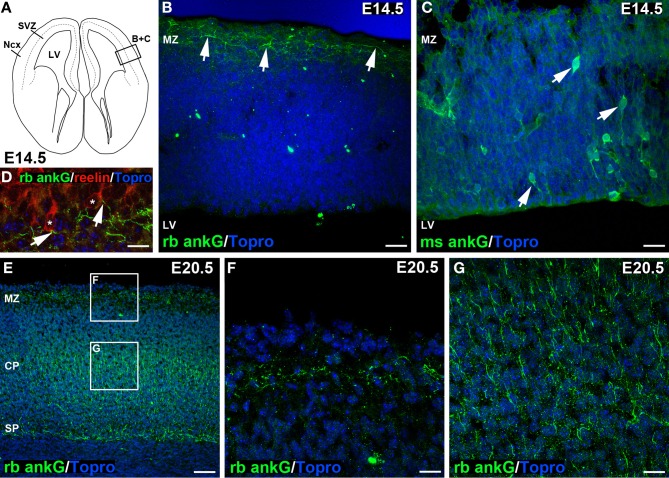
**Distinct ankG signal in embryonic sections at E14.5 and E20.5. (A)** Line graph of an E14.5 coronal section of mouse brain, modified from Schambra et al. (1991). Box indicating **(B)** + **(C)** highlights region of interest depicted in **(B,C)**. **(B)** Distinct ankG immunosignal at E14.5 resulting from polyclonal rabbit antibody (green) was localized to AIS of putative Cajal-Retzius cells in the marginal zone (MZ, arrows), also shown in **(D)**. AIS appear predominantly horizontal along the pial surface of the cortex. **(C)** Somatodendritic ankG immunosignal resulting from usage of monoclonal mouse antibody (green) at E14.5. Cells appear mostly bipolar with processes extending from the soma (arrows). No AIS were observed at E14.5 with this antibody. **(D)** Reelin-positive Cajal-Retzius cells (red, soma delineated by asterisk) with ankG (green) along their putative AIS (arrows) in a predominantly horizontal orientation in an E14.5 brain. **(E)** Overview of E20.5 cortex showing rabbit anti ankG immunoreactive AIS (green) with distribution along the MZ, a broad band within the cortical plate (CP) and subplate (SP). **(F)** Magnification of upper box in E indicating AIS in the MZ at E20.5 in horizontal orientation. **(G)** Magnification of lower box in E with rabbit anti ankG-positive AIS (green) in the CP in predominantly perpendicular orientation. LV, lateral ventricle; Ncx, Neocortex; (S)VZ, (sub)ventricular zone; MZ, marginal zone; CP, cortical plate; SP, subplate. Scale bars **(A,B)** = 35 μm, **(D)** = 10 μm, **(E–G)** = 25 μm, **(F)** = 50 μm.

### AIS first appear during the embryonic period of corticogenesis

During cortical development, the first AIS appeared at E14.5 (Figures 2A–C). Only few scattered neurons in the marginal zone already exhibited an AIS (Figure 2B, arrows). At this age, marginal zone neurons harbored numerous mainly horizontally orientated ankG-positive processes, which seemed to arise from reelin-positive somata (Figure 2D, arrows). Reelin is an extracellular matrix protein characteristic for Cajal-Retzius cells, which are transient neurons with a small horizontal axon plexus in layer I of the developing cortex (Frotscher et al., 2009; Meyer, 2010). A polyclonal rabbit anti-ankG antibody raised against a 214 amino acid sequence in the C-terminus of ankG stained AIS specifically in the marginal zone, and also in cell populations residing in the cortical plate and subplate at E20.5 (Figures 2E–G). On consecutive sections incubated with a monoclonal mouse anti-ankG antibody directed against a fusion protein of 934 amino acid length, we found a subset of neurons throughout the cortex displaying cytosolic ankG immunoreactivity along bipolar processes and in the soma (Figure 2C, arrows). This somatic ankG staining was no longer observed at E20.5. From the beginning of the postnatal period throughout adulthood, both the polyclonal and monoclonal antibody gave identical signals (AIS only; Figures S1A–F). Generally, the orientation of AIS showed a distinct difference when comparing cells in the marginal zone with cells in the cortical plate and subplate. At E14.5 through E20.5, AIS of neurons in the marginal zone were predominantly horizontal (Figures 2E,F), while AIS in the cortical plate and subplate at E20.5 and later were mostly perpendicular (Figures 2E,G).

### AIS undergoes dynamic length maturation during the postnatal period

The dynamic maturation of AIS in visual cortex, area 17 in layers II/III and V was investigated by immunofluorescence staining and confocal microscopy in combination with AIS length measurements based on fluorescent pixel intensity.

**Figure 3:**
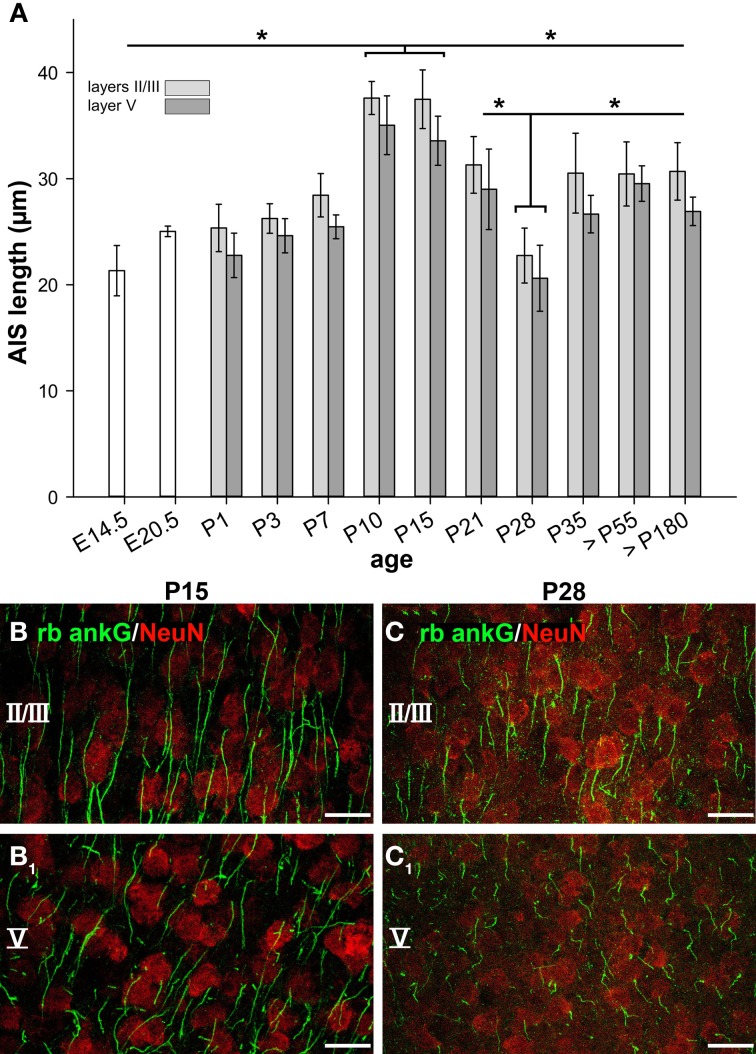
**AIS length maturation during visual cortex development. (A)** The first AIS appeared at E14.5 often associated with Cajal-Retzius cells. AIS in presumptive cortical neurons increased in length until a maximum from P10-P15. Subsequently, a significant AIS length shortening occurred from the beginning to the peak of the CP in mice (P19–28). Beginning with the closure of the CP at P35, AIS length stabilized, persisting throughout adulthood (>P180). Similar observations were made for layer II/III and layer V neurons, with layer V AIS showing overall shorter length. **(B)** Longest AIS expansion in layer II/III neurons at P15 as indicated by ankG (green) and somatic NeuN (red). **(B**_1_**)** Layer V image from the same cortical section as in **(B)**. **(C**_1_**)** Significant AIS length shortening in layers II/III in a P28 animal as compared to AIS length shown in **(B)**. Layer V image from the same cortical section as in **(C)** with shorter AIS. Scale bars **(B–C**_1_**)** = 25 μm. ^*^p ≤ 0.05, standard deviation.

As illustrated in Figure 3A, AIS significantly increased in length from embryonic to early postnatal ages. At E14.5 and E20.5, the single bars represent neurons from the entire cortical plate, since cortical layers are not yet reliably discernible. At P1 and P3, layer V is well discernible. Values presented as layers II/III in these two age groups represent neurons of the cortical plate. A sharp increase in AIS length from P7 on resulted in maximum values at P10–15 (37.59 ± 1.56 μm, layers II/III, Figures 3B,B_1_). The values differed significantly from the younger ages for both layers. Equally surprising was the sharp decline in AIS length in both layers until P28 (22.75 ± 2.58 μm, layers II/III, Figures 3C,C_1_) which corresponds to the maximum of the CP in mice. Since we did not observe a gap between the AIS and the soma, the shortening must have occurred from the distal end. AIS length then re-increased in the older age groups to a median of 30.5 μm at P35 up to >P180. Interestingly, mice of the C57BL/6JRj strain open their eyes between P13 and 14 (our own observations), which coincides with the end of the developmental peak at P15, suggesting that the significant AIS length reduction observed after P15 was triggered by the onset of vision. The tri-phasic developmental profile as detailed above was observed for neurons in cortical layers II/III and V in an identical manner. Moreover, it is evident that the average AIS length of layer V neurons was always trailing that of layers II/III neurons.

**Figure 4:**
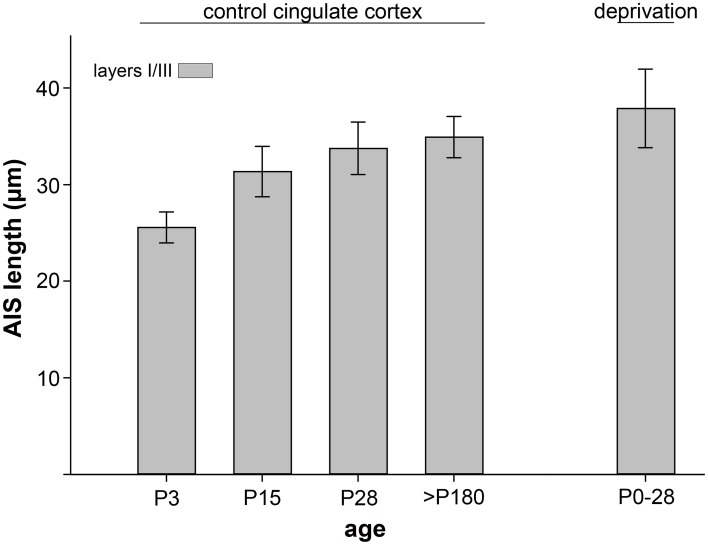
**AIS length maturation in a non-visual, non-sensory cortical region, the cingulate cortex**. AIS length of layer II/III neurons increased moderately during postnatal development and reached mature measurements after the peak of the CP (P28). No significant (*p* > 0.1) tri-phasic length maturation was observed in at least 100 AIS per cortex in 3 animals. As a control for dark-rearing experiments, the far right bar indicates no significant length changes after a 4 week deprivation period.

To test whether the observed tri-phasic AIS length maturation is a function of visual cortex development, a non-visual and non-sensory cortex, the cingulate cortex, was analyzed in a control experiment. AIS length analysis at the most important points of the tri-phasic development, namely P3, P15 (maximum length), P28 (minimum length) and >P180 (adult) showed that AIS fail to undergo significant changes (*p* = 0.1, Wilcoxon rank sum test). As outlined in Figure 4, AIS length increased during the postnatal period and remained rather stable throughout adulthood.

### ankG protein expression appears to be stable during late cortical development

**Figure 5:**
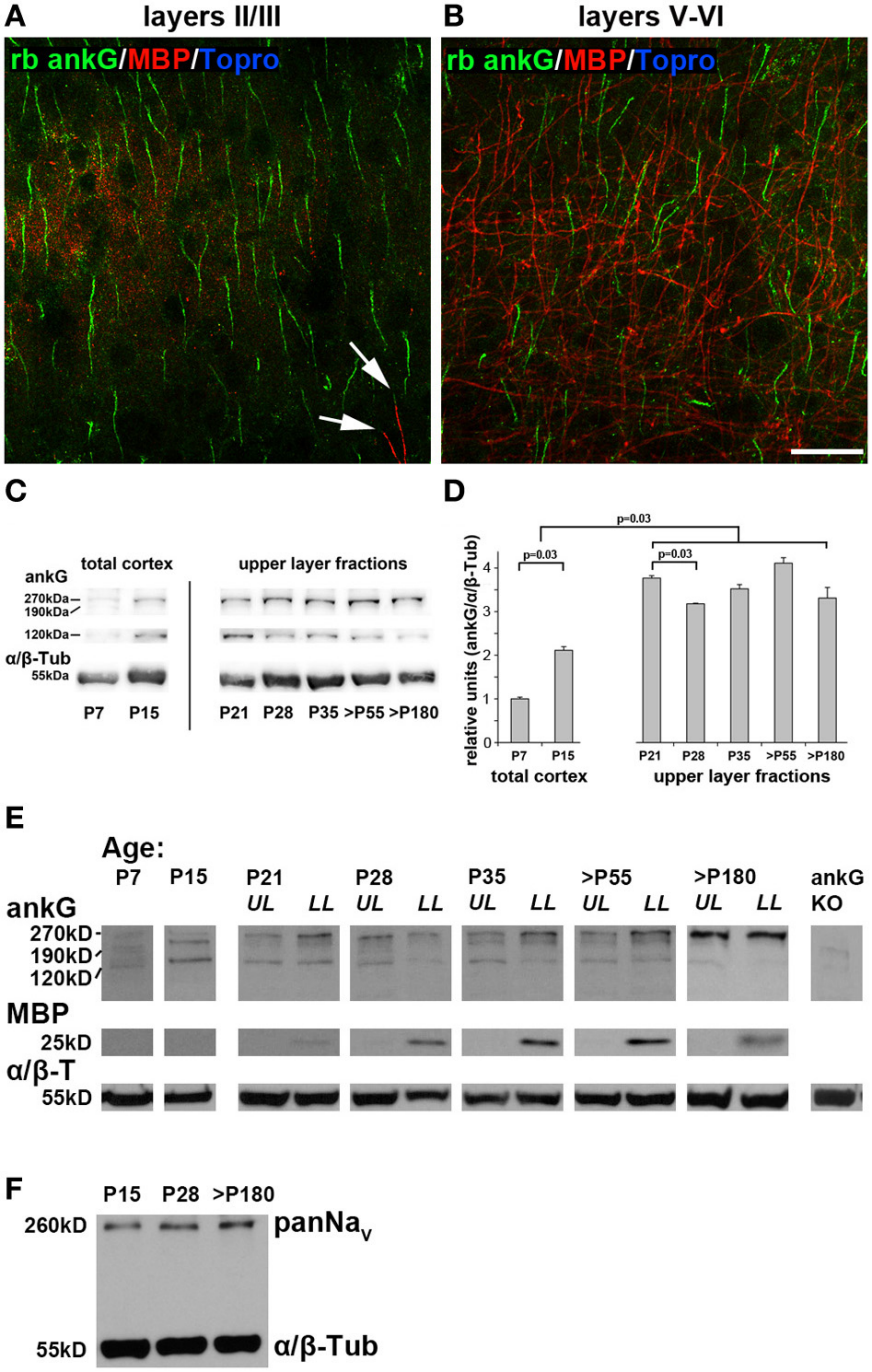
**AnkG, myelin and voltage-gated sodium channels (panNaV) protein expression during AIS development in visual cortex. (A)** Myelin distribution in layers II/III in an adult mouse (P67) with ankG (green) and myelin basic protein (MBP, red). MBP immunosignals were only rarely observed (arrows). **(B)** Myelin distribution in layers V and VI in the same section as **(A)**. MBP is more evident in infragranular than supragranular layers. **(C)** Representative immunoblot bands for ankG and α/β tubulin in total cortex samples from P7 and P15, and upper layer lysates from P21 through >P180 ages. **(D)** Quantification of ankG expression at various time points during development. P7 and P15 bars represent total visual cortex lysates, ages P21 and older the upper layer fractions only. AnkG protein was normalized to the internal loading control (α/β Tubulin) and levels are expressed relative to the normalized ankG expression at P7, which was set to 1. Shown are bars with standard deviation from three replicates. AnkG expression increased significantly during postnatal development (P7–21). From P21 to 28, coinciding with the maximum of length reduction (Figure 3A), ankG protein was reduced. However, despite significant re-elongation of the AIS past P28 (Figure 3A), no significant changes in protein amount were detected (*p* ≥ 0.05). **(E)** Representative immunoblot bands for ankG, MBP, and α/β tubulin in various age samples from P21 through >P180 were separated for upper and lower cortical layers (UL, upper layers; LL, lower layers). P7 and P15 samples were obtained from whole visual cortex lysates. MBP first appeared around P21. Far right lane shows immunoblot from a cerebellar ankG knockout mouse confirming specificity of rabbit anti ankG antibody. **(F)** PanNa_*V*_ immunoblot from the three stages of the tri-phasic AIS length development shows constant levels for sodium channel expression despite significant AIS length changes (Figure 3A). Scale bars **(A,B)** = 25 μm.

Next, we addressed if AIS maturation corresponds to changing amounts of ankG protein. Notably, ankG is not only expressed in the AIS, but also in nodes of Ranvier (Salzer, 2003). Myelination increases with the onset of the CP and is a driving force for its closure (McGee et al., 2005). Interestingly, supragranular layers of area 17 show little to no myelination (Figure 5A). In contrast, layers V and VI are heavily myelinated and therefore contain higher amounts of nodal ankG and myelin (Figure 5B). Therefore, care was taken to generate layer-specific protein samples for immunoblot analysis. Using this approach, potential contamination from myelin and nodal ankG of the white matter in supragranular samples could be eliminated.

Layer-specific Western blot analysis revealed a significant increase of ankG protein from P7 to 21 (Figures 5C,D). The myelin basic protein (MBP) band was clearly present in lysates from infragranular layers, but barely detectable in the supragranular fractions beginning at P21 (Figure 5E). We concluded that myelin and thus also nodal ankG were almost absent in upper layers suggesting that the increase of ankG protein in layer II/III fractions can largely be attributed to AIS development. At P28, when AIS show significant length shortening, ankG protein amount is also reduced (Figure 5D). However, at all later stages, a fairly constant level of ankG protein expression was observed, despite significant re-elongation of AIS length (Figures 3A, 5D). For infragranular layers, a constant level was expected because AIS shrinkage and presumed decrease of ankG in this microdomain might have been masked by an increase of nodal ankG. However, the results obtained in supragranular layers might suggest a local compression of ankG or dynamic assembly and disassembly mechanisms at the AIS.

As described above, after P28, AIS length re-increased to a mature length that is maintained throughout adulthood. It is higher than the P28 minimum, but lower than the P10/15 peak values, which might represent the physiological extremes of AIS length extension (Figure 3A). Immunoblots showed fairly stable ankG expression from P28 to 180 (Figures 5C,D). Interestingly, Western blot analysis of voltage-gated sodium channels (panNa_*V*_) expression showed a stable expression pattern with no decrease at P28 (Figure 5F). Taken together, these data might imply that a dilution and compression via lateral molecular movements of ankG and its binding partners in the membrane, or partial disassembly of the ankG/βIV spectrin membrane scaffold complex cause the AIS length changes.

### Sensory deprivation prevents AIS maturation in visual cortex

**Figure 6:**
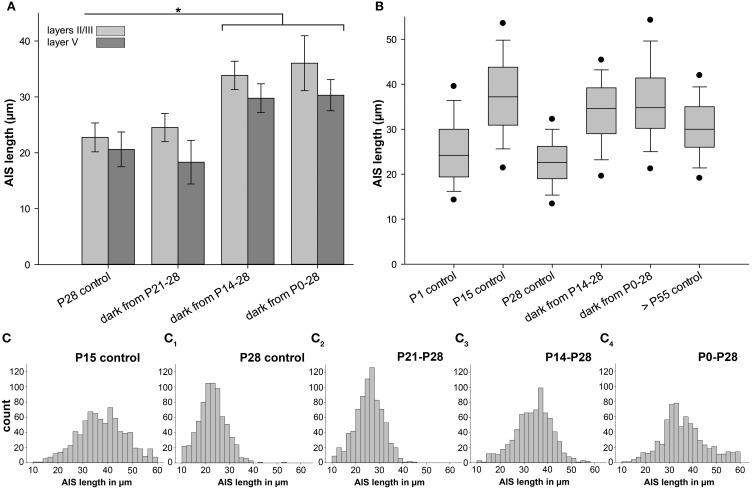
**Changes in AIS length after sensory deprivation**. Graph in **(A)** shows results for neurons in layer II/III (light gray) and V (dark gray); all other graphs indicate layer II/III data only. **(A)** Mice were placed in complete darkness from P21 to 28, showing no change compared to P28 control. Sensory deprivation from P14 to 28 resulted in a significant AIS length increase compared to P28 control. Likewise, dark rearing from birth to P28 resulted in a significant AIS length increase compared to P28 control. **(B)** Box plot comparison of AIS length percentiles and median in each experimental group revealed that juvenile AIS at P15 and before have a wider distribution of individual lengths. At the age of shortest AIS length (P28), AIS length distribution is the narrowest. Dark rearing from birth to P28 results in a similar length distribution as in juvenile AIS at P15. Adult AIS (>P55) show less width in length distribution, but are not as homogeneous as AIS at P28, the phase of highest compression. Vertical bars indicate upper 10% and lower 90% distribution, dots upper 5% and lower 95% percentile. Horizontal bars delineate the median. **(C–C**_4_**)** Size frequency histograms of various age groups under control and sensory deprivation conditions. **(C)** Wide distribution of AIS lengths at the peak of juvenile AIS length increase at P15. **(C**_1_**)** Narrow AIS length distribution at P28 when AIS are shortest. **(C**_2_**)** Sensory deprivation for 1 week and after eye opening has no effect on AIS length distribution compared to P28 controls. **(C**_3, 4_**)** Under sensory deprivation for at least 2 weeks, mature AIS had a broader length distribution and resembled the developmental P15 peak as shown in **(C)**. ^*^p ≤ 0.05, standard deviation.

The observed tri-phasic remodeling of AIS length (Figure 3A) in visual cortex, and the lack of this dynamic regulation in non-visual cortex (Figure 4), suggests that visual activity after eye opening is an important factor for AIS development. We therefore tested if the loss of visual input has an impact on AIS maturation. Placing mice in complete darkness for various periods deletes visual input but conserves intrinsically generated network activity. In a first set of experiments, mice were placed in darkness for 1 week from P8 to 15 and P21 to 28. No change of AIS length was observed (layers II/III P8–15 dark: 41.86 ± 2.58 μm vs. P15 control: 37.48 ± 2.76 μm; Figure 6A: P21–28 dark: 24.51 ± 2.53 μm vs. P28 control: 22.75 ± 2.58 μm). Next, mice were raised in darkness from P0 to 15. Again, AIS length was not altered compared to P15 controls (layers II/III P0–15 dark: 37.96 ± 1.44μm vs. P15 control: 37.48 ± 2.76 μm).

Mice then underwent sensory deprivation from P21 to 35 and from P0 to 35. Compared to P35 controls, which are significantly longer during normal development than the P28 minimum AIS length, neither group showed significantly changed AIS length (layers II/III P35 control: 30.52 ± 3.76 μm; P21–35 dark: 30.42 ± 1.94 μm; P0–35 dark: 33.32 ± 3.60 μm). However, when comparing different deprivation time windows in the P28 group, striking results were obtained (Figure 6A). In mice deprived for 2 weeks from P14 to 28 and 4 weeks from P0 to 28, a significant AIS length increase was observed (Figure 6A). The average AIS length in deprived animals was almost identical to the peak length at P10/15 (layers II/III P14–28 dark: 33.85 ± 2.53 μm vs. P10 control: 37.59 ± 1.56 μm; layers II/III P0–28 dark: 36.02 ± 4.92 μm vs. P10 control: 37.59 ± 1.56 μm; compare Figures 3A, 6B). In the control situation in non-visual cortex, no significant changes in the P0–28 dark-reared group could be observed (Figure 4; non-visual cortex layer II/III P28 33.75 ± 9.57 vs. P0–28 dark 37.89 ± 11.07). These data suggest that vision-evoked developmental changes of the cortical network during the CP cause the transient reduction in AIS length. Possibly, if the developmental peak at P15 is not terminated by the onset of vision, AIS remain in their extended, juvenile state, representing immature AIS.

Length frequency histograms revealed that juvenile AIS at P10/15 had a wider distribution than AIS at P28, and after 1 week of visual deprivation (Figures 6C–C_2_). Strikingly, AIS from 2 week as well as 4 week sensory-deprived animals also displayed the juvenile length distribution (Figures 6C_3_,C_4_). The broader distribution indicates an increased heterogeneity of AIS length maturation under these conditions. Adult AIS (>P55) became more uniform in length and showed less scatter in distribution, but they were not as uniform as AIS at P28 (Figure 6B).

## Discussion

**Figure 7:**
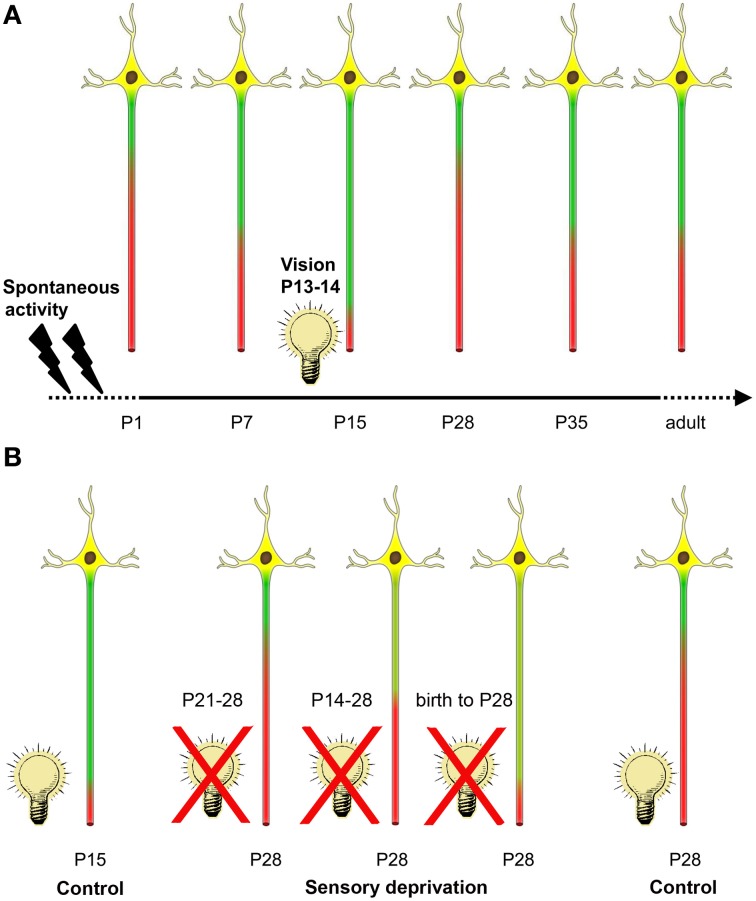
**Summary of activity-dependent AIS maturation in visual cortex. (A)** Spontaneous activity in form of retinal waves and intrinsic network activity along the developing visual system is present during the embryonic and early postnatal period (indicated by flashes). AIS length maturation is characterized by a tri-phasic time course of structural remodeling. First, during the early postnatal period, AIS steadily increase in length until a maximum is reached at P15. Coincidentally, between P13–14, mice of this strain open their eyes and receive visual input for the first time (indicated by light bulb). Shortly after eye opening, a striking AIS length shortening occurs. After the peak of the critical period at P28, AIS undergo another phase of length remodeling and increase to maintain mature length throughout adulthood. **(B)** A 1 week sensory deprivation (P21–28) in mice that have undergone eye opening and therefore AIS shortening did not alter AIS length compared to controls at P28 (far right neuron). However, a 2 week deprivation that began around the time of eye-opening (P14–28) was sufficient to prevent AIS length shortening compared to P28 controls. This effect was even more pronounced in dark reared (birth to P28) animals. Strikingly, this last deprivation group also showed similar AIS length distribution as the juvenile AIS group at P15 during normal development (far left neuron).

In the present study, a tri-phasic time course of AIS maturation in the developing visual cortex was observed (Figure 7A). Our data indicate that visual experience significantly influences the developmental reduction of AIS length, which coincides with the peak of the CP.

### Early appearance of AIS in visual cortex

We observed ankG-positive AIS as early as E14.5 in reelin-positive Cajal-Retzius cells of the marginal zone. These cells derive from the preplate, which has emerged about 2 days before (Hevner et al., 2003). Cajal-Retzius cells express sodium currents from E13 (Albrieux et al., 2004), are reported to fire APs (Sava et al., 2010), and are involved in the propagation of spontaneous activity (Aguilo et al., 1999). Likely, these functions require a mature AIS. Indeed, at E14.5, the polyclonal rabbit anti-ankG antibody revealed ankG at the AIS of marginal zone neurons. Immunoreactivity was neither in the soma nor along the distal axon, which could be interpreted as a second indicator of functional maturity. In early neurons, the entire cell bodies and neuritis can be ankG positive, and a recent study in embryonic mouse spinal cord motor neurons finds transient ankG expression along the entire axon and soma at E9.5 (Le Bras et al., 2013).

A peculiar observation was that staining with a mouse monoclonal anti-ankG antibody at this age labeled a select population of bipolar cells throughout the cortical plate. Only at postnatal stages did both antibodies label the AIS of cortical neurons in an identical manner. Non-specific staining or epitope sharing in a transient cell population may account for the observed discrepancies at that early state and further analysis is required in order to determine specificity of ankG antibodies at this developmental stage.

Our study suggests that marginal zone neurons require structurally and functionally intact AIS earlier than the co-generated presumptive subplate neurons, which had no AIS at E14.5. Instead, they displayed ankG-positive AIS at E20.5. The third cellular subset with ankG-positive AIS at this age were neurons in presumptive layer V. At E20.5, supragranular neurons and interestingly also layer VI neurons did not yet display ankG-positive AIS. By comparison, an earlier study conducted in motor cortex reported the expression of ankG and formation of the AIS in cortical plate neurons between E18 and birth (Galiano et al., 2012). This might be due to regional differences between the motor and visual cortex pointing to the diversity of AIS development in these distinct cortical areas.

### The length of AIS changes during postnatal development

From E20.5, we observed a continuous AIS length increase during the first postnatal week reaching maximum length values at P10. This length persisted during eye opening (P13–14 in mouse) until P15. In mouse motor cortex, a similar length increase from P1 to 5, albeit more pronounced, was observed previously (Galiano et al., 2012). Strikingly, we detected a significant AIS length reduction reaching a minimum at P28, the peak of the CP. At this time, AIS had become fairly uniform in length. Thereafter, the average length re-increased to a level in-between the P15 maximum and the P28 minimum. The changes occurred at the same time in supra- and infragranular neurons suggesting that it is not influenced by ontogenetic age. A similar alteration of AIS length has been observed during the development of monkey prefrontal cortex with a significant length reduction from postnatal to adult stages (Cruz et al., 2009; Fish et al., 2013).

AIS length frequency histograms revealed that a substantial number of AIS with a length of 50 μm or longer were present in early postnatal neurons. Mature AIS displayed a narrower scatter; they become more homogeneous in length. This underlines the significant heterogeneity and potential plasticity of the postulated intra-axonal barrier governing AIS length (Galiano et al., 2012). A substantial reorganization of molecular AIS components has been reported for somatic motoneurons, which establish the mature AIS and the AP initiation zones toward the beginning of the myelin sheath over the first two postnatal weeks (Duflocq et al., 2011). In our study, the maximum AIS length was reached at P10, 3–4 days before eye opening, eliminating visual input as a prerequisite for early AIS length increase. Indeed, visual deprivation from P8 to 15 did not induce AIS length changes when compared to P15 controls. Thus, it seems that the continuous increase from late embryonic to P10/P15 occurs either cell-autonomously or, alternatively, under the contribution of spontaneous activity. Retinal waves, for instance, are essential for proper visual circuit assembly (Huberman et al., 2008) and drive early patterns of primary visual neocortical activity (Hanganu et al., 2007). In the mouse, retinal waves emerge around E16 (Bansal et al., 2000), and cease around the time of eye opening (Huberman et al., 2008). It is therefore possible that intrinsic retinal activity triggers an AIS length increase in early postnatal visual cortical neurons.

Interestingly, AIS length analysis at the critical ages of P7, P15 (maximum AIS length), P28 (minimum AIS length) and >P180 (adult AIS length) in a non-visual cortical region, the cingulate cortex, showed no significant tri-phasic length maturation at all. AIS from that same region in dark-reared animals (birth to P28) were also not affected. This could suggest that sensory cortical regions may undergo a fundamentally different AIS maturation than non-sensory areas, which might not require the same levels of activity in order to form their mature connections.

### Functional implications of AIS maturation

Our ankG staining's show a dynamic remodeling of AIS length. Since ankG serves as the master scaffolding protein of the AIS, it is tempting to speculate that molecular components of the AIS linked to ankG show similar patterns and dynamic expression profiles with direct implication for neuronal excitability. In particular, the distribution and clustering of sodium channels at the AIS is directly linked to the targeting of ankG protein to this compartment (Zhou et al., 1998; Garrido et al., 2003). Studies in the chick auditory system suggest that longer AIS render neurons more excitable (Kuba et al., 2010). After auditory deprivation and loss of presynaptic input, neurons of the chick nucleus magnocellularis increase their AIS length 1.7-fold and become more excitable (Kuba et al., 2010). In line with this, elongated AIS result in a heightened state of excitability in a pathological mouse model of Angelman Syndrome (Kaphzan et al., 2011). Correlating our data, we suggest that cortical networks consisting of neurons with long AIS at P10/P15 acquire a heightened state of excitability. Indeed, the higher excitability of supragranular neurons at P10/P15 has been attributed to the formation of exuberant horizontal connections and synaptogenesis (Li et al., 2010). Moreover, an increased NMDA receptor-mediated excitatory activity has been observed for cortical neurons from P11-P20, a period of increased susceptibility for epilepsy which ends around P28 (Luhmann and Prince, 1990). Concurrently, between P10 and 15 a specific enrichment of Na_*V*_1.6 and K_*V*_ channels occurs at the AIS (Liao et al., 2010), which might have direct consequences for AP firing. Indeed, rat visual cortical neurons aged P9–13 (with long AIS) display slower onset dynamics for APs and are barely able to encode frequencies >50 Hz (Ilin et al., 2013). By contrast, at P17–30 (during and after AIS shortening) the neurons become able to encode high frequencies and to strongly phase-lock to a stimulus (Ilin et al., 2013). It seems plausible that the increase in AIS length, the molecular maturation of the AIS, and the increased network excitability during and shortly after eye opening, are processes which re-enforce each other in a positive manner.

Our data suggest that eye opening and visual activity during the precritical period drive AIS length maturation, including the shortening of the AIS. The P15 and P28 AIS lengths from the present study might represent physiological extremes for sensory cortical AIS development. The mature AIS length distribution likely represents the functional optimum. Going through both extremes during normal development might enable neurons to “learn” about the physiological range within which the AIS length could be up- or downscaled in order to up- or downscale neuronal excitability. This raises the fundamental question whether the underlying mechanisms represent a scaling parameter of homeostatic plasticity. Homeostatic plasticity has been postulated for synaptic changes in the somatodendritic domain of neurons (Turrigiano, 2012). The significant developmental elongation and compression of the AIS in visual cortex occurred around 1 week before opening and closing, respectively, of the CP. Since AIS length changes influence excitability and AP firing (Kuba, 2010), it should be taken into consideration that maximum and minimum AIS length might also be necessary to prepare the cortical network for the opening and subsequent closure of the CP.

### Factors influencing AIS maturation

Proof for the role of sensory experience for AIS maturation was obtained by our dark-rearing experiments (Figure 7B). Strikingly, the compression of AIS length was absent when mice were kept in darkness from P14 to 28, or were dark-reared from birth until P28. Furthermore, length frequency histograms from these experimental groups showed the broad scatter typical for AIS during the juvenile length peak at P10/P15. Therefore, we suggest that visual deprivation delays AIS maturation.

Dark-reared cortical neurons display decreased excitation by visual stimuli, strong habituation, and low spontaneous discharge rates (Fagiolini et al., 1997). Excitatory synapses of dark-reared cortical neurons at P20–24 and P50 have the low AMPA/NMDA receptor ratio normally seen at eye opening (Funahashi et al., 2013). Not only AMPA receptor currents remain low, also NMDA receptor currents are impaired in dark-reared cortex (Funahashi et al., 2013). Dark rearing also impairs the development of GABAergic inhibition. Usually, between eye opening and the closure of the CP in normal rats, the total GABAergic input converging into layer II/III pyramidal neurons increases significantly (Morales et al., 2002). Although GABA receptor expression is barely influenced by dark-rearing (Heinen et al., 2004), GABAergic synapses have functional deficits in release probability (Morales et al., 2002). This has been correlated with a failure of the GABAergic system to close the CP (Hensch, 2005). The AIS in dark-reared cortical neurons stay at the juvenile length distribution and fail to shorten on time. With respect to the physiological deficits of visually deprived neurons, this can be interpreted as an attempt to preserve the juvenile level of excitability. Accordingly, neurons maturing with normal visual experience will initiate an AIS shortening right at the time when intrinsically generated spontaneous activity patterns become replaced by visual input. Now, excitation is relatively high whereas inhibition is still relatively immature which results in a higher susceptibility for epilepsy (Luhmann and Prince, 1990). The AIS length changes reported in our study could lower the excitability of pyramidal cells. This could be neuroprotective and help to consolidate neuronal networks. Around the time when visual plasticity has come to an end and when the powerful GABAergic synaptic mechanisms have been fully implemented, the AIS re-elongate to the adult optimum, a level intermediate between the P15 maximum length and the P28 minimum length.

Other potential factors that might have an impact on AIS maturation are myelination and cortical innervation patterns. Myelination ascends from the white matter and is most prominent in infragranular layers (Jhaveri et al., 1992; Antonini et al., 1999). Recently, the interaction of myelin with the extracellular matrix has been implicated as a determining factor of node of Ranvier assembly (Galiano et al., 2012). It remains unknown if a similar mechanism applies to the AIS. We observed a steady increase in myelin in immunoblots from visual cortex at postnatal ages, and a constant expression throughout adulthood despite the structural AIS length compression after the onset of vision. Moreover, murine visual cortex myelination starts after P18 (McGee et al., 2005), which is after the AIS compression has been initiated. Thus, myelination is rather unlikely to influence the AIS length shortening and re-elongation.

Cortical innervation patterns could also be implicated in AIS length maturation. Pyramidal cell receive GABAergic synapses from axo-axonic chandelier neurons, which form toward the distal end of the AIS (Howard et al., 2005). In monkey prefrontal cortex the shortening of the AIS during postnatal development from 3 to 7 months of age coincides with a decline in the number of chandelier cell buttons per AIS (Fish et al., 2013). Thus, a link between AIS length alteration and chandelier cell axon terminal maturation might occur in a subset of visual cortical neurons in the present study. However, we have shown that AIS length changes occur in supra and infragranular layers. Yet, chandelier cells occur predominantly in supragranular layers (Peters et al., 1982; Meyer and Ferres-Torres, 1984). Therefore, it is unlikely that axo-axonic innervation is a driving force of the observed AIS length maturation in visual cortex.

Taken together, our results suggest that AIS plasticity is essential to generate the AP initiation site of mature neurons. The tri-phasic AIS maturation corresponds to important developmental steps of cortical network formation and is regulated by visual experience.

### Conflict of interest statement

The authors declare that the research was conducted in the absence of any commercial or financial relationships that could be construed as a potential conflict of interest.

## Supplementary material

Figure S1 **Specificity analysis for two commercially available ankG antibodies, polyclonal rabbit anti-ankG and monoclonal mouse anti-ankG (see Table 2) in postnatal visual cortex. (A)** Rb anti-AnkG (green) and ms anti-ankG (red) in layer II/III neurons in a P15 animal. Signal overlap is indicated by yellow staining. **(B)** Fluorescence intensity profile for both antibodies for the representative AIS from **(A)** indicated by an arrow. Graph shows signal overlap for both antibodies. **(C)** Same as **(A)**, but in P28 visual cortex. **(D)** Same as **(B)**, but for the AIS indicated by an arrow in **(C)**. **(E)** Same as **(A)**, but in >P180 visual cortex. **(F)** Same as **(B)**, but for the AIS indicated by an arrow in **(E)**. Scale bars **(A,C,E)** = 10 μm.

Figure S2 **Specificity analysis for two commercially available antibodies, a polyclonal rabbit anti-βIV spectrin and a monoclonal mouse anti-ankG (see Table 2) to show that the ms ankG antibody specifically stains AIS in postnatal tissue. (A)** Ms anti-AnkG (green) and rb anti- βIV spectrin (red) in layer II/III neurons in a P15 animal. Signal overlap is indicated by yellow staining. **(B)** Fluorescence intensity profile for both antibodies for the representative AIS from **(A)** indicated by an arrow. Graph shows signal overlap for both antibodies. **(C)** Same as **(A)**, but in P28 visual cortex. **(D)** Same as **(B)**, but for the AIS indicated by an arrow in **(C)**. **(E)** Same as **(A)**, but in >P180 visual cortex. **(F)** Same as **(B)**, but for the AIS indicated by an arrow in **(E)**. Scale bars **(A,C,E)** = 10 μm.
