# Supplementary material for: A period of structural plasticity at the axon initial segment in developing visual cortex
Source: Front Neuroanat. 2014 Mar 11;8:11. doi: 10.3389/fnana.2014.00011 (PMC3949221; doi:10.3389/fnana.2014.00011)
Supplement: Figure S1 — Specificity analysis for two commercially available ankG antibodies, polyclonal rabbit anti-ankG and monoclonal mouse anti-ankG (see Table 2) in postnatal visual cortex. (A) Rb anti-AnkG (green) and ms anti-ankG (red) in layer II/III neurons in a P15 animal. Signal overlap is indicated by yellow staining. (B) Fluorescence intensity profile for both antibodies for the representative AIS from (A) indicated by an arrow. Graph shows signal overlap for both antibodies. (C) Same as (A), but in P28 visual cortex. (D) Same as (B), but for the AIS indicated by an arrow in (C). (E) Same as (A), but in >P180 visual cortex. (F) Same as (B), but for the AIS indicated by an arrow in (E). Scale bars (A,C,E) = 10 μm. [file Presentation1.PDF]

Supplementary Material

A period of structural plasticity at the axon initial segment in developing visual cortex

Annika Gutzmann[1], Nursah Ergül[1], Rebecca Grossmann[1], Christian Schultz[1],Petra Wahle[2], Maren Engelhardt[1*]

[1]Institute of Neuroanatomy, CBTM, Medical Faculty Mannheim, Heidelberg University, Germany
[2]AG Developmental Neurobiology, Faculty of Biology and Biotechnology, Ruhr-University, Bochum, Germany

*** Correspondence:** Maren Engelhardt, Institute of Neuroanatomy, CBTM, Heidelberg University, Ludolf-Krehl-Str. 13-17, 68167 Mannheim, Germany
maren.engelhardt@medma.uni-heidelberg.de

Supplementary Data

We present supplementary data indicating specificity of two different, commercially available ankyrinG antibodies.

1. Supplementary Figures and Tables

We observed a significant change in staining patterns for a polyclonal rabbit anti-AnkG and a monoclonal mouse anti-ankG antibody when comparing embryonic and postnatal cortical samples. In embryonic stages, only the rabbit anti-ankG stains the AIS of cortical neurons, while the mouse anti-ankG stains a subpopulation of bipolar-appearing cortical cells (Fig. 2 in our manuscript). Here we prove that in the postnatal period, both antibodies stain the same structure, namely the AIS of cortical neurons.

1.1. Supplementary Figures

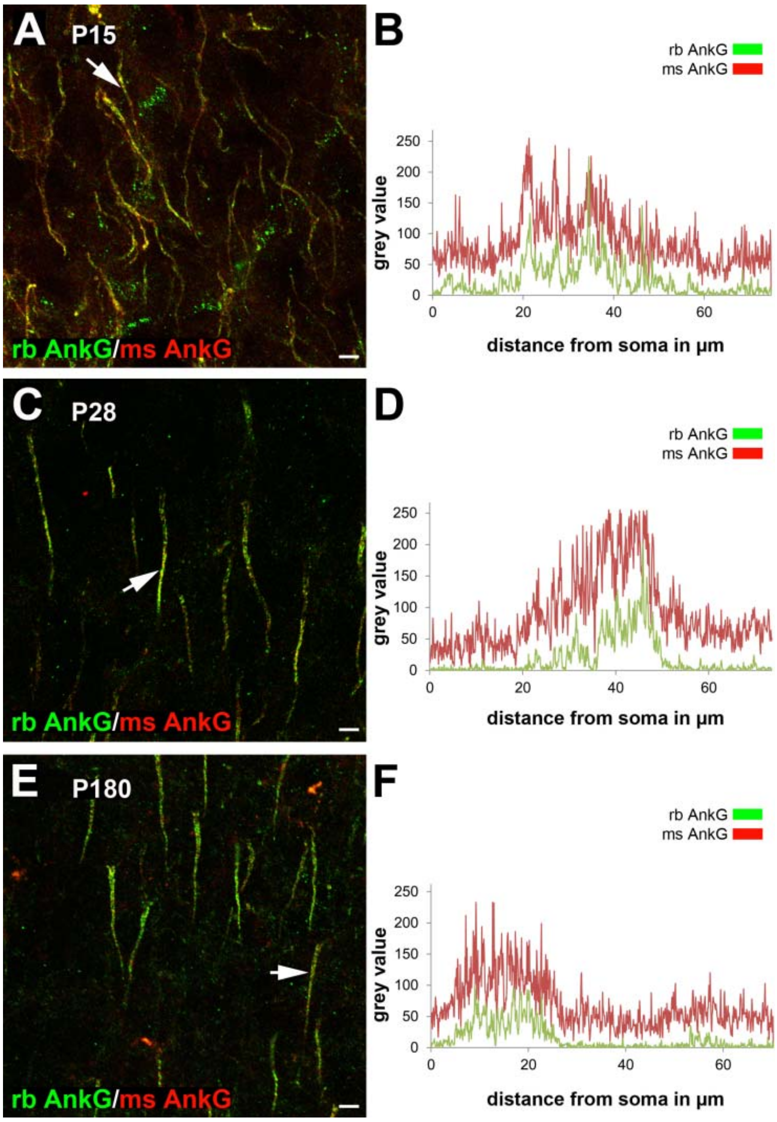

Supplementary Figure S1. Specificity analysis for two commercially available ankG antibodies, polyclonal rabbit anti-ankG and monoclonal mouse anti-ankG (see Table 2) in postnatal visual cortex. **(A)** Rb anti-AnkG (green) and ms anti-ankG (red) in layer II/III neurons in a P15 animal. Signal overlap is indicated by yellow staining. **(B)** Fluorescence intensity profile for both antibodies for the representative AIS from (A) indicated by an arrow. Graph shows signal overlap for both antibodies. **(C)** Same as (A), but in P28 visual cortex. **(D)** Same as (B), but for the AIS indicated by an arrow in (C). **(E)** Same as (A), but in >P180 visual cortex. **(F)** Same as (B), but for the AIS indicated by an arrow in (E). Scale bars A, C, E = 10 µm.

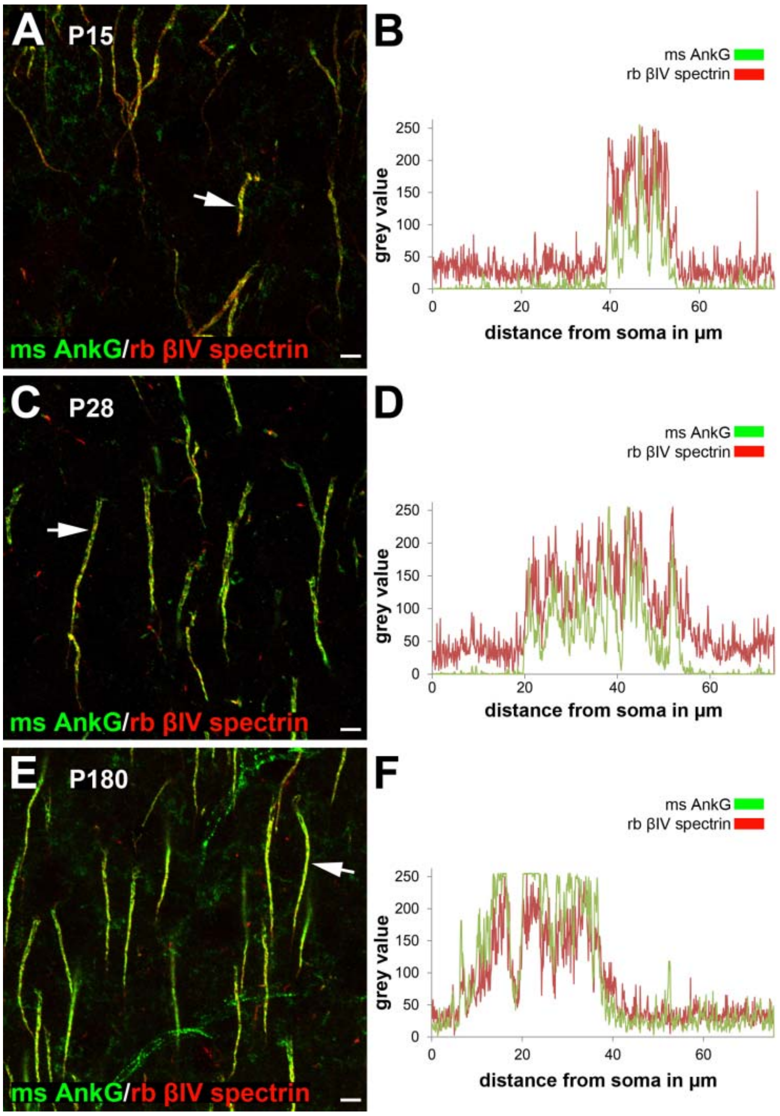

Supplementary Figure S2. Specificity analysis for two commercially available antibodies, a polyclonal rabbit anti-βIV spectrin and a monoclonal mouse anti-ankG (see Table 2) to show that the ms ankG antibody specifically stains AIS in postnatal tissue. **(A)** Ms anti-AnkG (green) and rb anti- βIV spectrin (red) in layer II/III neurons in a P15 animal. Signal overlap is indicated by yellow staining. **(B)** Fluorescence intensity profile for both antibodies for the representative AIS from (A) indicated by an arrow. Graph shows signal overlap for both antibodies. **(C)** Same as (A), but in P28 visual cortex. **(D)** Same as (B), but for the AIS indicated by an arrow in (C). **(E)** Same as (A), but in >P180 visual cortex. **(F)** Same as (B), but for the AIS indicated by an arrow in (E). Scale bars A, C, E = 10 µm.
